# Investigation of the effects of arch size and implant angulation on the accuracy of digital impression using two intraoral scanners: An in vitro study

**DOI:** 10.1002/cre2.793

**Published:** 2023-10-03

**Authors:** Farideh Geramipanah, Leyla Sadighpour, Leila Payaminia

**Affiliations:** ^1^ Department of Prosthodontics, Faculty of Dentistry, Dental Implant Research Center Tehran University of Medical Sciences Tehran Iran

**Keywords:** accuracy, arch size, digital impression, implant

## Abstract

**Objectives:**

The aim of this in vitro study was to evaluate the effect of arch size and implant angulation on the accuracy of digital impression in two intraoral scanners of Trios (3shape) and CEREC (Omnicam).

**Material and Methods:**

Four acrylic models each including six implants at sites 11, 12, 15, 17, 23, and 27 were used, including large with parallel implants, large with angled implants, small with parallel implants, and small with angled implants. After tightening the scan bodies, distance measurements were done using a coordinate measuring machine. Then, each model was scanned 10 times using each scanner. Trueness and precision measurements were finally computed.

**Results:**

The trueness values ranged from 20 to 260 μm in CEREC Omnicam, and from 40 to 1030 μm in Trios. The precision values ranged from 30 to 190 μm in CEREC Omnicam, while from 50 to 770 μm in Trios. The multivariate test analysis indicated that the measured distances via two scanners and different models show different behaviors. Pairwise interactions between these three variables were significant (*p* < .05). Pairwise interactions between these variables were also significant. (*p* < .0001).

**Conclusions:**

Arch width could affect the accuracy of digital impression; by rotating toward the second quadrant and end points of the scan, errors have increased. However, the angulation of the implants had no effect on the accuracy of digital impression. The CEREC Omnicam scanner showed higher accuracy (trueness and precision) compared to the Trios (3shape) one.

## 1 INTRODUCTION

The passive fit is considered as an essential factor for long‐term predictability of an implant‐supported prosthesis. A highly accurate transfer of the position and direction of implants to the master cast is a key step to ensure a passive fit (Di Fiore et al., 2015; Lee et al., 2008). Otherwise, an inaccurate impression may lead to misfits and consequently potential mechanical and biological complications (Di Fiore et al., 2015; Eliasson et al., 2010; Lee et al., 2008).

Impressions could be made by either conventional or digital techniques. Both techniques have their inherent advantages and drawbacks. The advantages of digital impressions include simplicity, patient comfort concerning the taste and setting time of impression material particularly for patients with high gag reflexes, no need to remake the whole impression in case of unclear parts within it, reduced time, less distortion caused by impression materials, easy and rapid data transfer and no risk of model breakage while transferring (Christensen, 2009; Giménez et al., 2015a; Goracci et al., 2016; Grünheid et al., 2014; Jansen et al., 1997; Joda & Brägger, 2016; Yuzbasioglu et al., 2014).

Several studies have found similar fit in single dental (Boeddinghaus et al., 2015; Zarauz et al., 2016)/implant (Delize et al., 2019; Lee et al., 2015) crowns made by digital impressions compared to conventional counterparts. However, for full‐arch reconstructions, studies still suffer from conflicting results. Some have demonstrated insignificant difference between intraoral digital and conventional impressions (Papaspyridakos et al., 2016). Several of them have reported superior outcomes in digital techniques (Alikhasi et al., 2018). Contrarily, others believed that conventional impressions are more accurate (Ender & Mehl, 2013, 2015; Giménez et al., 2014, 2015b). Inter‐implant distance and implant angulation in multiple‐unit reconstructions seem to be effective factors on the accuracy of intraoral digital impression making.

Moreover, arch size affects inter‐implant distance in different dimensions (anterior–posterior and cross‐arch). It has been reported that arch size has no significant influence on the accuracy of the conventional full arch implant impressions (Rezaei et al., 2021). Although, in the case of digital impressions, it has been shown that errors increase with an increment in the scanning distance (Ciocca et al., 2018; Di Fiore et al., 2019), the effect of arch size has not been clearly investigated.

Angulated implants are inevitably a prevalent problem due to anatomical limitations and clinician factors (Chia et al., 2017). Various studies have shown that angulated implants may lead to inaccurate conventional impressions (Akalin et al., 2013; Mpikos et al., 2012; Rezaei et al., 2021; Rutkunas et al., 2012; Sorrentino et al., 2010). However, a few studies have concluded that implant angulation does not significantly affect the accuracy of digital impressions (Giménez et al., 2015a, 2015b). However, due to the intrinsic difference of conventional and digital impression techniques, more evaluations are needed for digital impressions.

The aim of the present study was to evaluate the effects of arch size and implant angulation on the accuracy of digital impressions using two intraoral scanners of Trios (3shape) and CEREC (Omnicam). The null hypotheses were the claim that 1—arch size, 2—implant angulation, and 3—type of intraoral scanner do not affect the digital impression accuracy.

## 2 MATERIALS AND METHODS

### 2.1 Ethical statement

The present in vitro study was based on acrylic models; accordingly, no Ethics Committee approval nor consent to participate was requested for this research.

**Figure 1:**
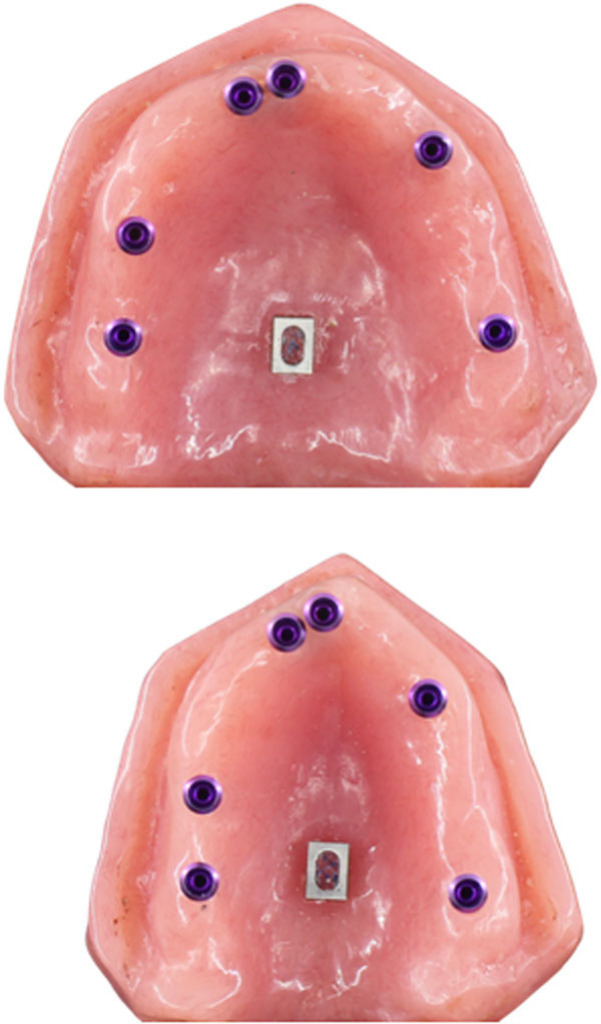
Resin models with the implants inserted.

A large‐sized cast of an edentulous patient who was adapted to the largest stock tray and a small‐sized one which was adapted to the smallest stock tray were used to fabricate the examined models. Each was duplicated twice in acrylic resin. The inter‐canine and inter‐molar distances in the large model were considered as 35 and 50 mm, respectively, while being 25 and 40 mm in small model, respectively. The process was suspended for a week to ensure that the resin polymerization process is completed, and models are dimensionally stable. After 1 week, a hole was made in each model using a CNC machine, in accordance with the diameter and length of the implants and the desired angle. In each model, six Straumann regular neck tissue level implants (Institute Straumann AG) were placed at sites 11, 12, 15, 17, 23, and 27 (FDI System). These sites were considered to simulate different numbers of pontics in fixed dental prosthesis. In a pair of models (one large and one small model), implants were inserted parallel to each other, while in the other two ones, the angle of implant insertion was 30° mesially at sites 27 and 17, 0° (parallel) at sites 23 and 15, and 15° buccally at sites 11 and 12. In addition, in the middle of the palate of each model, a rectangular aluminum block with dimensions of 5 mm × 7 mm with a round slot in the center (in the form of two semicircles with a diameter of 1.5 mm and a 2 mm spacing) was used as a reference so that its upper plan was completely parallel to the lower surface of the model (see Figure 1). Finally, the implants and reference blocks were fixed in place using auto‐polymerizing resin.

**Figure 2:**
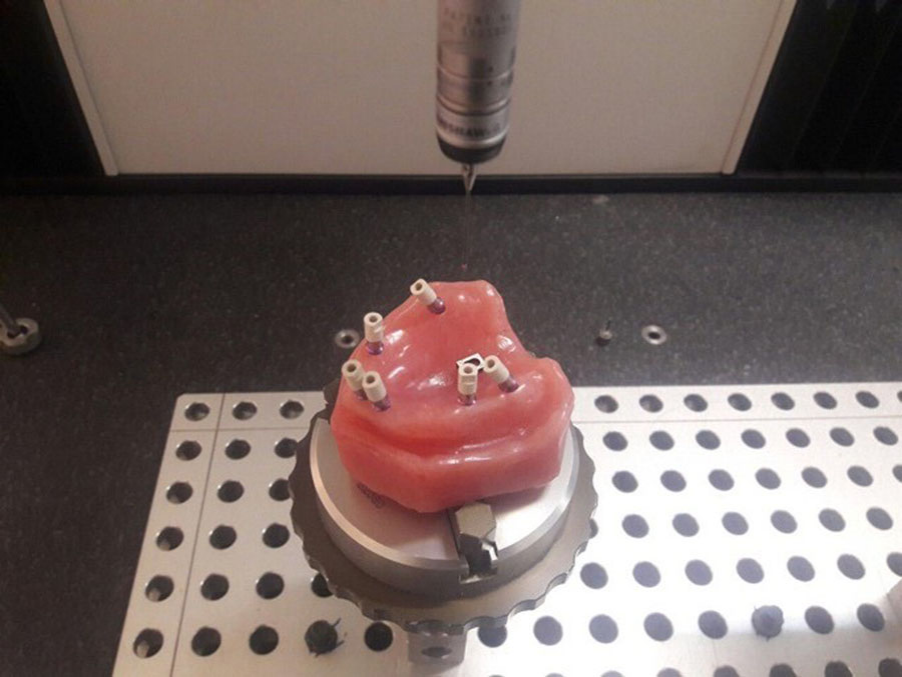
Distance measurements between the center of scan bodies using a coordinate measuring machine (CMM).

The Straumann tissue‐level Core Scan bodies (Straumann tissue‐level, RC [compatible], Core3D Centers) were connected to the implants and tightened with 10 N cm torque according to the manufacturer's instructions. To achieve the accurate measurements, the distances between the scan body at site 27 and each of the scan bodies at sites 11, 12, 15, 17, and 23 in each model were measured using a coordinate measuring machine (CMM) with an accuracy of 0.0028 mm on the *X*–*Y*–*Z* axes, and analyzed with its contact probe (see Figure 2).

Digital impression was made by a trained operator after learning curve completion (16 scans) by means of two intraoral scanners of Trios cart scanner v. 1.4.7.5 (Trios® 3 basic Cart wired, 3shape) and CEREC Omnicam v 4.3 (Sirona Dental Systems). Each model was scanned 10 times by each scanner; therefore, a total of 80 digital impressions were obtained. The scanning procedure was started from the reference block in the palate, followed by the palatal surfaces from site 27 to 17, occlusal surfaces from site 17 to 27, and finally buccal surfaces from site 27 to 17. Care was taken to record the connection and middle areas of each scan body well. After performing the evaluation process, unclear points were re‐scanned. Then, using the postprocessing option in the scanner systems, the overall scan was corrected.

The output files of CMM in Initial Graphics Exchange Specification (IGES or IGS) format and the scan files in Standard Tessellation Language (STL) format were transferred to Geomagic qualify 12 software. To evaluate the accuracy of the scanners, trueness (the agreement between actual and scanned sizes) and precision (the closeness of the scan sizes to each other within a group using a scanner) were computed.

### 2.2 Trueness measurement

**Figure 3:**
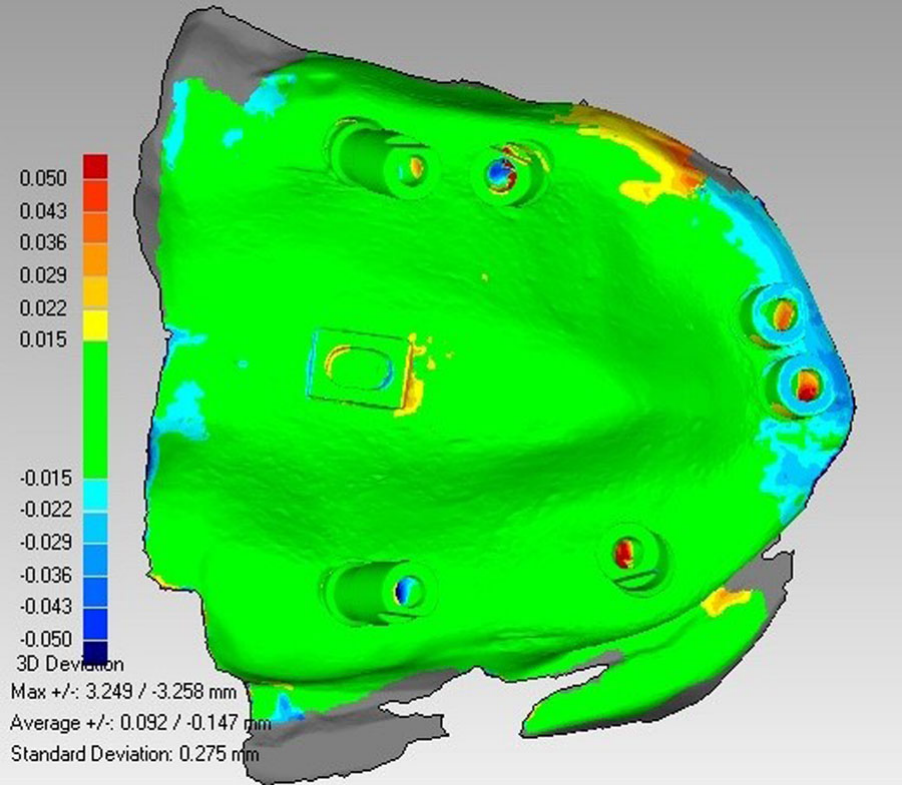
Color graph of the superimposed files.

In the software environment, an IGS and an STL file were selected as the reference and test files, respectively. The best‐fit algorithm was also used to superimpose them. To do this, the number of common points was considered to be 2000 points. Then, the three‐dimensional compare analysis was performed. As a result, a color graph was generated with minimum and maximum values of −50 to +50 μm, which represented the quantitative differences between the two superimposed files. The green color represented the chosen tolerance scale (−15 to +15 μm), while warm colors indicated a positive difference (outward) of the test file compared to the reference file in the range of +15 to +50 μm. Furthermore, cool colors indicated a negative difference (inward) of the test file compared to the reference file in the range of −15 to −50 μm (Figure 3).

**Figure 4:**
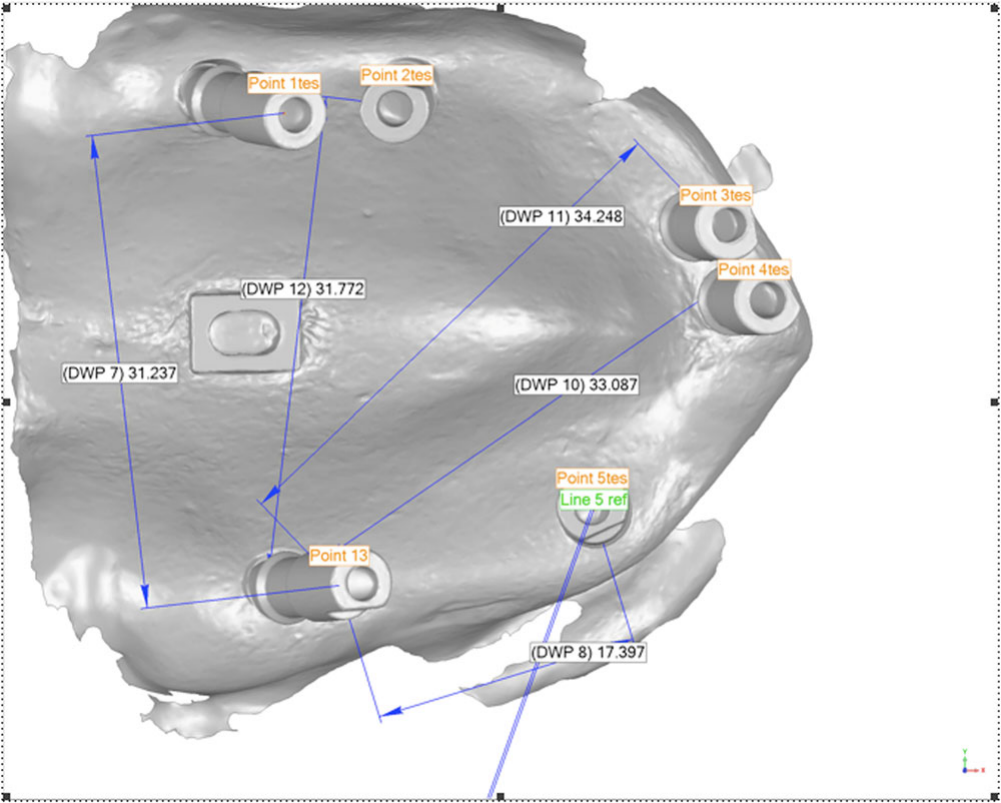
Distance measurements between the center of scan body 27 and those of scan bodies 11, 12, 15, 17, and 23.

**Figure 5:**
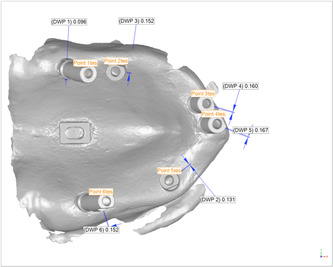
Displacement measurements: the differences between the centers of each scan body in the reference (CMM file) and the test (scan files) files.

For the samples in which all implants were parallel, the “plan section to object” option was used. The origin of coordinates was placed in the center of the reference block, and a section was passed through the center of the scan bodies at a point 16 mm above the origin in the *Z*‐axis (vertical). To make a comparison, this section was considered the same in all files. For samples in which the implants were angled, first of all, the axis of each scan body was determined, and then, a section was made at the level of the parallel scan bodies from the centers. In the acquired section, the distances between the center of the scan body 27 and those of the scan bodies 11, 12, 15, 17, and 23 were measured (Figure 4). In addition, the differences between the center of scan bodies in the reference and test files indicating the amount of displacement were measured (Figure 5). The reason for choosing scan body 27 as the reference of the measurements was due to the scan strategy used in the present study, which started from the mentioned scan body.

### 2.3 Precision measurement

To measure the precision, the scan file with the best trueness value in each group was selected as the reference file, and other scans as the test files were then superimposed to that. The next steps were performed in the same manner as described in the trueness determination procedure.

### 2.4 Statistical analysis

Data analysis was performed using SPSS version 23. To determine descriptive statistics, mean and standard deviation measurements were calculated for dependent variables. Two‐way analysis of variance was also employed to conduct statistical analyses for independent variables. Due to significant interactions, the relationship between independent and dependent variables was analyzed in a pairwise manner in study subgroups. The level of significance (*p* value) was set at .05.

## 3 RESULTS

### 3.1 Trueness

Mean and standard deviations of distance variation and displacement variables between the test and the reference files corresponding to the four examined models (large with parallel implants, large with angled implants, small with parallel implants, and small with angled implants) scanned by CEREC Omnicam and Trios (3Shape) scanners are summarized in Table 1. As would be observed, the trueness values range from 20 to 260 μm in CEREC Omnicam, while from 40 to 1030 μm in the case of Trios.

**Table 1:** Trueness values.

		CEREC Omnicam		Trios (3Shape)	
Model	Measurements	Mean	SD	Mean	SD
Large with parallel implants	D1	0.05	0.02	0.18	0.15
	D2	0.05	0.03	0.04	0.04
	D3	0.05	0.04	0.05	0.04
	D4	0.12	0.07	0.78	0.58
	D5	0.26	0.15	0.56	0.32
	∆27	0.09	0.03	0.19	0.05
	∆23	0.02	0.01	0.52	0.14
	∆11	0.05	0.02	1.01	0.07
	∆12	0.04	0.04	1.03	0.07
	∆15	0.06	0.08	0.63	0.26
	∆17	0.12	0.13	0.43	0.32
Large with angled implants	D1	0.05	0.04	0.12	0.02
	D2	0.10	0.05	0.27	0.36
	D3	0.10	0.07	0.23	0.27
	D4	0.07	0.07	0.46	0.39
	D5	0.14	0.06	0.13	0.09
	∆27	0.04	0.04	0.31	0.65
	∆23	0.05	0.03	0.64	0.79
	∆11	0.04	0.03	0.36	0.67
	∆12	0.12	0.09	0.37	0.64
	∆15	0.14	0.23	0.70	0.13
	∆17	0.16	0.23	0.59	0.35
Small with parallel implants	D1	0.07	0.05	0.14	0.10
	D2	0.08	0.05	0.11	0.08
	D3	0.05	0.04	0.07	0.13
	D4	0.08	0.06	0.25	0.11
	D5	0.08	0.08	0.20	0.07
	∆27	0.05	0.03	0.08	0.06
	∆23	0.03	0.04	0.17	0.05
	∆11	0.07	0.04	0.20	0.30
	∆12	0.07	0.04	0.18	0.31
	∆15	0.08	0.04	0.34	0.14
	∆17	0.07	0.04	0.27	0.16
Small with angled implants	D1	0.04	0.03	0.15	0.12
	D2	0.08	0.06	0.22	0.37
	D3	0.13	0.09	0.18	0.32
	D4	0.09	0.07	0.17	0.06
	D5	0.07	0.07	0.16	0.05
	∆27	0.14	0.12	0.15	0.19
	∆23	0.13	0.10	0.12	0.16
	∆11	0.14	0.17	0.19	0.19
	∆12	0.13	0.17	0.15	0.18
	∆15	0.14	0.07	0.05	0.03
	∆17	0.15	0.15	0.06	0.05

*Note*: D1: The distance difference between the centers of scan body 23 and 27 in the scan file with the mentioned distance in CMM file. D2: The distance difference between the centers of scan body 11 and 27 in the scan file with the mentioned distance in CMM file. D3: The distance difference between the centers of scan body 12 and 27 in the scan file with the mentioned distance in CMM file. D4: The distance difference between the centers of scan body 15 and 27 in the scan file with the mentioned distance in CMM file. D5: The distance difference between the centers of scan body 17 and 27 in the scan file with the mentioned distance in CMM file. ∆27: The difference between the centers of scan body 27 in the scan and CMM files. ∆23: The difference between the centers of scan body 23 associated with the scan and CMM files. ∆11: The difference between the centers of scan body 11 in the scan and CMM files. ∆12: The difference between the centers of scan body 12 in the scan and CMM files. ∆15: The difference between the centers of scan body 15 in the scan and CMM files. ∆17: The difference between the centers of scan body 17 corresponding to the scan and CMM files.

In general, for both CEREC Omnicam and Trios (3Shape) scanners, the best trueness was observed in the distances between scan bodies 27 to 23 and 11 (first quadrant or near to midline) and the worst one was observed in the distances between scan bodies 27 to 15, 12, and 17 (second quadrants). Moreover, the highest displacement error was observed in scan bodies 15, 17, and 12, while the least error value was recorded for scan bodies 23, 27, and 11.

### 3.2 Precision

**Figure 6:**
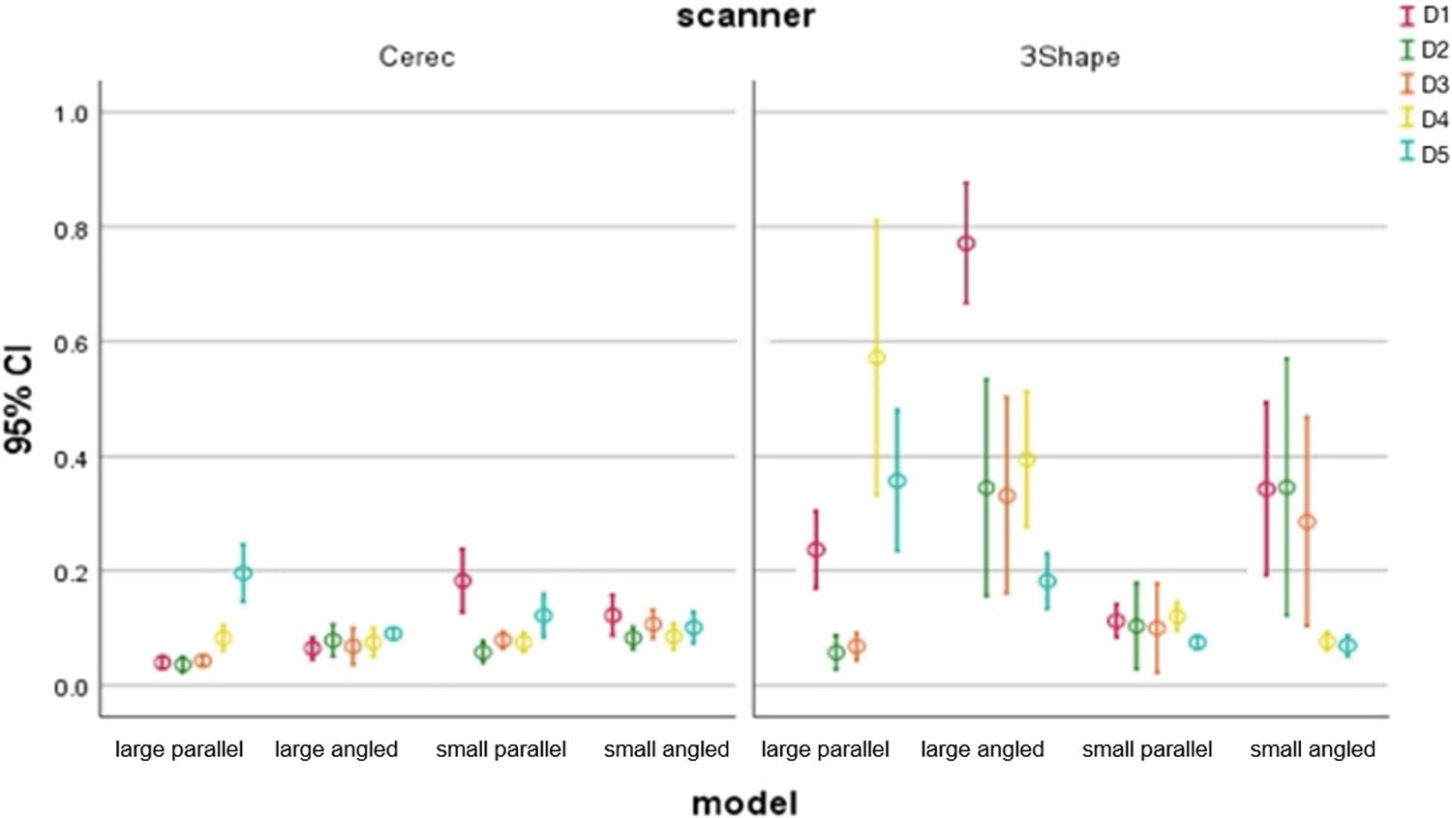
Precision in D1–5 distances of Trios (3shape) and CEREC (Omnicam) in four models: large with parallel implants, large with angled implants, small with parallel implants, and small with angled implants.

Mean and standard deviations of distance differences between the test and the reference files of four models (large with parallel implants, large with angled implants, small with parallel implants, and small with angled implants) scanned by CEREC Omnicam and Trios (3Shape) scanners are summarized in Table 2 and Figure 6. It was found that the precision values range from 30 to 190 μm for CEREC Omnicam and from 50 to 770 μm for Trios.

**Table 2:** Precision values.

		CEREC Omnicam		Trios (3shape)	
Model	Measurements	Mean	SD	Mean	SD
Large with parallel implants	D1	0.03	0.01	0.23	0.08
	D2	0.03	0.01	0.05	0.03
	D3	0.04	0.01	0.06	0.03
	D4	0.08	0.02	0.57	0.31
	D5	0.19	0.06	0.35	0.15
Large with angled implants	D1	0.06	0.02	0.77	0.13
	D2	0.07	0.03	0.34	0.24
	D3	0.06	0.04	0.33	0.22
	D4	0.07	0.03	0.39	0.15
	D5	0.09	0.01	0.18	0.06
Small with parallel implants	D1	0.18	0.07	0.11	0.03
	D2	0.05	0.02	0.10	0.09
	D3	0.07	0.01	0.10	0.10
	D4	0.07	0.02	0.12	0.03
	D5	0.12	0.04	0.07	0.01
Small with angled implants	D1	0.12	0.04	0.34	0.19
	D2	0.08	0.02	0.34	0.29
	D3	0.10	0.03	0.28	0.23
	D4	0.08	0.03	0.07	0.02
	D5	0.10	0.03	0.06	0.02

*Note*: D1: The distance difference between the centers of scan body 23 and 27 in the scan file with the mentioned distance in CMM file.  D2: The distance difference between the centers of scan body 11 and 27 in the scan file with the mentioned distance in CMM file. D3: The distance difference between the centers of scan body 12 and 27 in the scan file with the mentioned distance in CMM file. D4: The distance difference between the centers of scan body 15 and 27 in the scan file with the mentioned distance in CMM file. D5: The distance difference between the centers of scan body 17 and 27 in the scan file with the mentioned distance in CMM file.

In fact, the precision value corresponding to CEREC Omnicam was better than that of Trios (3Shape) scanner. No significant difference was found between the four models. Furthermore, the best precision was observed in small models with parallel implants scanned by 3shape scanner, and it decreased in large models with parallel implants.

The results via multivariate test analysis showed that the distances measured via two scanners and different models follow different trends. Pairwise interactions between these three variables were found to be significant (*p* < .05). According to the two‐way analysis of variance analysis, model and scanner variables were significantly effective on the distances D4 and D5, and on the displacements ∆11 and ∆12. Pairwise interactions between these variables were also significant (*p* < .0001).

The D4 distance associated with a large model with parallel implants significantly showed a larger distance error compared to the small ones, which was also larger in a 3shape scanner (*p* < .05).

D5 distance associated with a large model with parallel implants significantly showed a larger distance error compared to the other counterparts, which was also larger in a 3shape scanner (*p* < .05).

It was found a significantly large displacement error for scan bodies 11 and 12 in a large model with parallel implants rather than other models, which was also larger in a 3shape scanner (*p* < .05).

## 4 DISCUSSION

The present achievements rejected the first null hypothesis, implying that the arch width does not affect the accuracy of digital impression. The results showed that the maximum distance error was observed between the scan body 27 and those of 15 and 17 (in the contralateral quadrants) in large models. Also, the largest displacement errors were recorded for the scan bodies 15, 12, 11, and 17 (rotation toward the second quadrant and end points of the scan). These results are in line with those reported in the literature. Ciocca et al. (2018). showed that the errors increased with an increasing length of scan within the arch. Flügge et al. (2016). also reported that the precision of the intraoral scanning system decreases with an increasing distance between scan bodies. The errors occurred between the start and end points of the scan led to the highest errors at the endpoints (first molar regions). In a study conducted by Giménez et al. (2015b), in which an edentulous master model with six implants was scanned by CEREC Bluecam system (based on active triangulation technology), although the accuracy of digital impressions for the first scanned quadrant was high, it decreased when scanning a full arch. In addition, Ender and Mehl (2013) demonstrated the largest deviations in the second molar region, being at the distal end of the dental arch. The long distance between the implants interferes with the correct stitching of the images due to lack of reference points in the smooth surface of the mucosa (Giménez, Pradíes, et al., 2015; Rhee et al., 2015). In the present study, a reference block was placed in the middle of the palate to better stitch the images scanned by the scanner. In clinical practice, it is recommended to use pressure indicating paste (PIP), zinc oxide eugenol (ZOE), or flowable composite as a marker in palate or long‐distance edentulous areas (Fang et al., 2018).

The present results confirmed the second null hypothesis implying that the implants angulation has no effect on the accuracy of digital impression. Many studies have also confirmed that angulation has no significant effect on the accuracy of digital impressions (Alikhasi et al., 2018; Amin et al., 2017; Giménez et al., 2014, 2015b; Papaspyridakos et al., 2016; Ribeiro et al., 2018), unlike conventional impressions (Alikhasi et al., 2018; Amin et al., 2017; Ribeiro et al., 2018; Semper et al., 2010). This might be due to the need for higher force to be applied during conventional impression removal with angulated implants (Alikhasi et al., 2018). However, there is no impression material in digital impression making, and angulated implants could be easily scanned by rotating the intraoral scanner tip. This finding is consistent with the result of both studies conducted by Gimenez et al., in which neither the angulation nor the implant depth was recognized to be important factors affecting the accuracy of the scanners significantly (Giménez et al., 2015a, 2015b). Basaki et al. (2017) also indicated that the angle of the implants has no significant effect on the accuracy of the final casts made by digital and conventional techniques. The study carried out by Chia et al. also claimed that by digital scanning, distance error and angular distortion of mesiodistal direction do not significantly increase by increasing inter‐implant angulation. However, angular distortion of buccolingual direction has significantly increased (Chia et al., 2017).

The results of the present study refuted the third null hypothesis, which implied that the type of intraoral scanner has no effect on the accuracy of the digital impression. CEREC Omnicam scanner showed higher accuracy in terms of both trueness and precision than Trios (3shape) one. However, this is inconsistent with the results of previous studies. Trios was shown to be more accurate than CEREC Omnicam in the investigations of Di Fiore et al. (2019) and Medina‐Sotomayor et al. (2019). Imburgia et al. (2017) did not observe any statistically significant difference between the accuracy of Trios and CEREC Omnicam scanners. The heterogeneous results can be explained by different methodologies.

In this study, the trueness of digital implant impression was found to range from 20 to 260 μm for CEREC Omnicam, and from 40 to 1030 μm for Trios. Also, the precision ranged from 30 to 190 μm for CEREC Omnicam, while varying between 50 and 770 μm in the case of Trios. The mentioned values associated with CEREC Omnicam are comparable to the trueness and precision ranges (7.6 to 731.7 and 15.2 to 204.2 μm, respectively) reported in a systematic review by Zhang et al. (2021). However, the values corresponding to Trios were out of those ranges. Considering 100 μm as clinically acceptable threshold of deviation (Andriessen et al., 2014), these error ranges are too large for full arch digital impressions for both intraoral scanners.

Among the novelties of the present investigation, one can refer to the use of models with different arch sizes, as well as the placement of implants with different angulations and distances to simulate 5‐, 4‐, 3‐, and 2‐unit restorations. CEREC Omnicam and 3shape scanners do not need contrast powder, and are suitable for edentulous area scanning, while in LAVA COS and 3M TrueDef scanner systems, powder must be used to scan glossy or translucent surfaces, which improves the reflection properties, leading to an increased accuracy of the scan. However, the powder thickness could affect the scan accuracy. The PEEK scan bodies used in this study had appropriate light reflection properties and resulted in accurate scanning (Van der Meer et al., 2012).

It should be noted that as the current research was an in vitro study, CMM was used to measure trueness. However, this method could not be applied in clinic. The possible clinical methods to assess passive fitness of the framework are visual inspection, checking for the balance, one‐screw test (Sheffield test), and screw resistance test (Rutkunas et al., 2020). Moreover, although the results of the present in vitro study were satisfactory, a possible limitation could be intraoral scanning complications in clinical conditions, like jaw movement, saliva, and interference of the scanner tip with buccal tissue and tongue. This is not far from the expectation that these factors could increase the error rate compared to scanning the model.

## 5 CONCLUSION

The impact of arch width on the accuracy of digital impression is considered as a limitation of the current study. However, the errors have increased by rotation toward the second quadrant and end points of the scan. Besides, angulation of the implants was found to have no effect on the accuracy of digital impression. Further to this, the CEREC Omnicam scanner showed higher accuracy (trueness and precision) compared to Trios (3shape) one.

## AUTHOR CONTRIBUTIONS

Farideh Geramipanah and Leyla Sadighpour designed and performed the experiments. Leyla Sadighpour analyzed the data. Farideh Geramipanah and Leila Payaminia interpreted the data. Leila Payaminia wrote and revised the manuscript in consultation with Feramipanah Geramipanah and Leyla Sadighpour.

## CONFLICT OF INTEREST STATEMENT

The authors declare no conflict of interest.

## Data Availability

All data underlying the results are available as part of the article and no additional source data are required.
